# Hartshorn Dangers

**Published:** 1858-10

**Authors:** 


### HARTSHORN DANGERS.

An interesting little child died the other day, by having been given a vial of hartshorn to play with. The stopper came out, and part of the fluid getting into the throat and nose, caused an inflammation, which was not subdued.

A gentleman, ten days since, entered our office in evident suffering, and in short, hasty words, exclaimed: "He liked to killed me." Some one, for a slight throat affection, had swabbed it out with a sponge charged with spirits of hartshorn. The almost instant irritation scarcely allowed him to swallow, breathe, or speak, without great discomfort. In a note received to day, he says: "Your remedies were potent; but like 'the nine,' I have neglected to return and give thanks. P. S.—Coffee is coffee, whether made in the 'Old Dominion' or anywhere else: bear witness, my trembling hands."

We might have inquired of our respected correspondent: What was his opinion of hartshorn? Will he, and must every body else discard it forever, because its injudicious use endangered his life? And as he is one of the few young men of promise who abides by the Scriptures, we suggest, that it is a duty and privilege to enjoy all the good things of this life; the authority being in the Divine intimation: "To use this world, as not abusing it." The hermit is but half a saint; as he who avoids the battle-field is but half a soldier. To shun temptation is well; but to live in the midst of it, and never yield to it, from stern principle—that is true nobility of soul. To use tea and coffee, and meats, which our observation demonstrates, give comfort, and strength, and nourishment; but to use them with moderation, and a manly self-denial, that is the freedom which makes us "free indeed;" but to injure the body, to undermine the health and intellect, by their brutish abuse, and then turn around and spitefully kick at them—that is simply childishness.

---

The following sentence from the *Home Journal* fills our whole soul with the beauty of its lovingness and truth. "We might explain many a coldness, could we look into the heart concealed from us; we should often pity where we hate; love, when we think we can never forget; admire, when we curl the lip with scorn and indignation."

